# Early Contextual Fear Memory Deficits in a Double-Transgenic Amyloid-*β* Precursor Protein/Presenilin 2 Mouse Model of Alzheimer's Disease

**DOI:** 10.1155/2017/8584205

**Published:** 2017-11-27

**Authors:** Yasushi Kishimoto, Kai Fukumoto, Mika Nagai, Ayaka Mizuguchi, Yuiko Kobashi

**Affiliations:** Laboratory of Neurobiophysics, Kagawa School of Pharmaceutical Sciences, Tokushima Bunri University, 1314-1 Shido, Sanuki, Kagawa 769-2193, Japan

## Abstract

Presenilin 1 and presenilin 2 (PS1 and PS2) play a critical role in *γ*-secretase-mediated cleavage of amyloid-*β* precursor protein (APP) and the subsequent generation of *β*-amyloid peptides. The purpose of the present study was to test whether PS2 mutation accelerates the onset of contextual fear memory deficits in a mouse model of AD that expresses a mutation (K670N/M671L) of the human APP with the Swedish mutation (Tg2576 mice). In the present study, an APP/PS2 double-transgenic mouse model (PS2Tg2576) was generated by crossbreeding transgenic mice carrying the human mutant PS2 (N141I) with Tg2576 mice. Contextual fear conditioning was tested in PS2Tg2576 mice aged 3, 4, 6, and 10–12 months. PS2Tg2576 mice showed a tendency of lower freezing behavior as early as 3 months of age, but significant memory impairment was observed from the age of 4 months. The cognitive impairment was more prominent at ages of 6 and 10–12 months. In contrast, Tg2576 mice aged 3 and 4 months exhibited successful acquisition of contextual fear learning, but Tg2576 mice aged 6 months or older showed significantly impaired fear memory. These results show that PS2 mutation significantly accelerates the onset of fear memory deficits in the APP AD model mice.

## 1. Introduction

Alzheimer's disease (AD) is a progressive neurodegenerative disorder characterized by neuropathological features, including abnormal deposition of *β*-amyloid (A*β*) peptides, intracellular neurofibrillary tangles, and neuronal death, in selective brain regions including the hippocampus and cortex [1, 2]. AD is the most common form of dementia and is clinically characterized by the progressive loss of cognitive abilities, particularly the retention of recently learned information [3–5]. Accumulation of A*β* peptides is considered a key event in the pathogenesis of AD; presenilin 1 (PS1) and presenilin 2 (PS2) play a critical role in the *γ*-secretase-mediated cleavage of amyloid precursor protein (APP) and consequently the generation of A*β* peptides [6–8]. A mutation in one of these 3 genes (*APP*,* PS1*, and* PS2*) accounts for approximately 5 percent of all AD. This subtype of AD is known as familial Alzheimer's disease (FAD) [9, 10].

To elucidate the molecular basis of pathological processes in AD, several mouse models of AD have been developed, and most of these models exhibit transgenic expression of one or more mutations found in FAD [10, 11]. These mouse models represent a powerful tool for investigating the molecular basis of cognitive decline associated with AD [12], and many behavioral paradigms have been applied to evaluate cognitive function in these model animals. Among the behavioral tasks, contextual fear conditioning, along with the Morris water maze (MWM) task, has been widely used as a hippocampus-dependent test. Contextual fear conditioning occurs when a context becomes associated with an aversive unconditioned stimulus (US) such as a foot shock [13, 14]. Indeed, this learning paradigm has been tested in many transgenic mouse models of AD, including APP transgenic mice (Tg2576 mice) [15, 16], PS1/APP double-transgenic mice [17, 18], and 3xTg-AD mice, which harbor mutant genes for human APP, PS1, and tau [19, 20]. However, there has been no report describing contextual and cued fear conditioning in PS2/APP double-transgenic mice. Indeed, only a few studies have evaluated the effect of* PS2* mutation on cognitive performance by using PS2/APP double-transgenic mice [21–23]. Thus, in the present study, we tested contextual fear memory in PS2/APP double-transgenic mice (PS2Tg2576 mice) to determine the effect of* PS2* mutation on cognitive ability in AD model mice. These mice were generated by crossbreeding transgenic mice carrying the human mutant* PS2* (N141I) with Tg2576 mice [22, 23]. We also analyzed spontaneous physical activity, including locomotor activity, in PS2Tg2576 mice by using a previously developed automated video analysis system [24, 25].

## 2. Materials and Methods

### 2.1. Animals

Tg2576 mice, APP/PS2 double-transgenic (PS2Tg2576) mice, and control wild-type mice from the same litter were used for all behavioral assays. Tg2576 mice (Tg(APPSWE)2576Kha), which express human *β*-amyloid precursor proteins (hAPP) containing the K670N and M671L mutations on a 129S6 genetic background, were purchased from Taconic Farms, Inc. (Hudson, NY, USA) [11]. PS2Tg2576 were generated by crossbreeding Tg2576 male mice with PS2M1 female mice. PS2M1 mice express human PS2 proteins containing the N141I mutation on a C57BL/6JJcl background (purchased from Immuno-Biological Laboratories Co, Ltd., Fujioka, Japan) [22, 23]. The* PS2* transgene is expressed under control of a ubiquitous CAGGS promoter consisting of a cytomegalovirus early enhancer coupled with the chicken *β*-actin promoter [22]. The mouse genotyping was performed using the polymerase chain reaction (PCR) method [22]. Mice were housed in a room with controlled humidity (55 ± 5% relative humidity) and temperature (24 ± 2°C), and a 12 : 12-h light-dark (LD) cycle with light from 9:00 a.m. to 9:00 p.m. All mice were given ad libitum access to food and water. All behavioral experiments were performed during the light phase of the LD cycle. All mice were maintained until they reached the age of 3, 4, 6, or 10–12 months, and each mouse was used only once in the behavioral test. There were approximately equal numbers of male and female mice. All animal procedures were approved by the Tokushima Bunri University animal ethics committee and were performed in accordance with the guidelines laid down by the National Institutes of Health (NIH, Bethesda, MD USA).

### 2.2. Spontaneous Physical Activity in the Home Cage

Spontaneous physical activity was assessed in the home cage, as previously described [24–28]. Mice aged 3, 4, 6, and 10–12 months were transferred to new home cages (21 × 31 × 12 cm), identical to their original cages, and they were video recorded over a period of 3 h, from 09:00 a.m. to 12:00 p.m. to exclude the effects of circadian rhythm [28]. A camcorder (NV-GS300; Panasonic Corporation, Tokyo, Japan) was mounted on a tripod that was angled perpendicular to the cage in order to provide a side view of the cage. The camera footage was transferred to and saved in a Dell computer with Magic TV software (I-O DATA DEVICE, Inc., Kanazawa, Japan). The video movie data were analyzed with the HomeCageScan system (CleverSys, Inc., Reston, VA, USA). Spontaneous locomotor activities, such as rearing and distance traveled, were evaluated.

### 2.3. Contextual Fear Conditioning

Conditioning was performed in 26 × 32 × 21 cm operant chambers (CleverSys' Operant Chamber) in sound-attenuating boxes (43 × 46 × 43 cm). The chamber was equipped with a light, a speaker, and a stainless-steel rod floor through which a foot shock could be administered. All stimuli were controlled by FreezeScan computer software (CleverSys). The conditioning procedure was performed as previously described [16, 27, 29]. Mice (*n* = 10 per each genotype) were trained and tested on 2 consecutive days. On day 1, training consisted of placing the mouse in a chamber, illuminating the stimulus and house lights, and allowing exploration for 3 min. After the exploration, a 2 s footshock [1.5 mA; US] was administered, and mice were removed from the chamber 30 s later. 24 hrs after the training, mice were returned to the same chambers in which training occurred, and freezing behavior was recorded for 3 min (test trial). Freezing was defined as the absence of any movement except that required for breathing. Freezing was measured using the FreezeScan video tracking system and software (CleverSys) [30, 31].

### 2.4. Statistical Analysis

Data obtained in the behavioral tests were analyzed with a 2-way (age × genotype) analysis of variance (ANOVA), which was followed by a post hoc Bonferroni or *t*-test, with the GraphPad Prism (GraphPad Software, Inc., San Diego, CA). All of the data are presented as mean ± SEM. Significance was assigned at *p* < 0.05.

## 3. Results

### 3.1. Evaluation of Spontaneous Home-Cage Behaviors in PS2Tg2576 Mice

**Figure 1:**
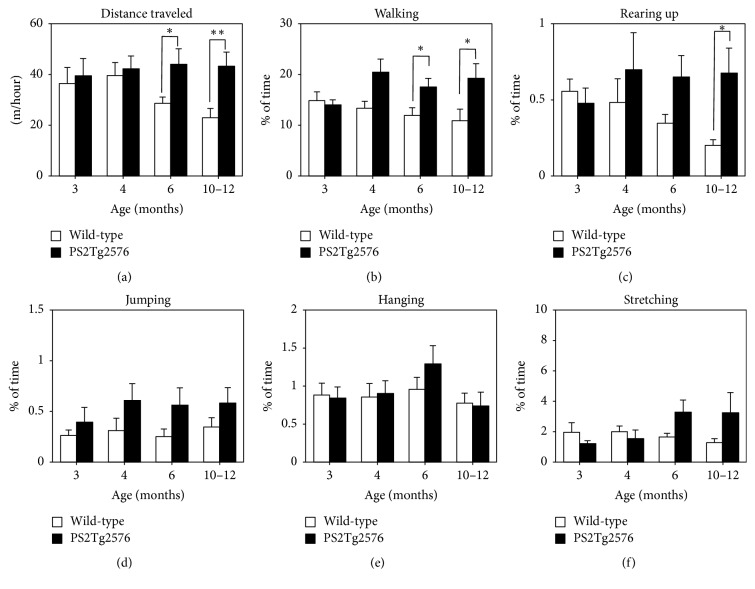
Spontaneous physical activities in PS2Tg2576 mice. The following 6 separate parameters of spontaneous behavior were evaluated in control wild-type mice (open column, *n* = 10) and PS2Tg2576 mice (closed column, *n* = 10) in the home-cage environment: (a) distance traveled, (b) walking, (c) rearing, (d) jumping, (e) hanging, and (f) body stretching. Ten mice of each genotype were used. The parameters were analyzed at ages of 3, 4, 6, and 10–12 months. At 6 months, PS2Tg2576 mice exhibited significantly higher spontaneous physical activities for distance traveled (a) and walking (b) (*p* = 0.03 and 0.025, resp.). At 10–12 months, PS2Tg2576 mice exhibited significantly higher spontaneous physical activities for distance traveled (a), walking (b), and rearing (c) (*p* = 0.0073, 0.036, and 0.011, resp.). Data points represent the mean ± SEM. ^*∗*^*p* < 0.05 and ^*∗∗*^*p* < 0.01 versus the corresponding control group.

First, we analyzed spontaneous physical activity by evaluating spontaneous behaviors in the home-cage environment during 4 stages of aging (3, 4, 6, and 10–12 months) (Figure 1). Six parameters of whole-animal movements, including distance traveled (Figure 1(a)), walking (Figure 1(b)), rearing (Figure 1(c)), jumping (Figure 1(d)), hanging (Figure 1(e)), and body stretching (Figure 1(f)), were calculated using HomeCageScan software. In control mice, slight decreases were observed in several physical activities (distance traveled and rearing) in an age-dependent manner (Figures 1(a) and 1(c)). In contrast, PS2Tg2576 mice exhibited no significant age-dependent alteration in any behavioral parameter. Although significant differences were not observed in any behavioral parameter between the 2 groups at ages of 3 and 4 months, statistically significant differences between the control and PS2Tg2576 mice were observed in 2 parameters (distance traveled and walking) at age of 6 months (Figures 1(a) and 1(c)). Furthermore, at 10–12 months, 3 parameters (distance traveled, walking, and rearing behavior) were significantly different between the control and PS2Tg2576 mice (Figures 1(a)–1(c)). In contrast, behaviors such as jumping, hanging, and stretching were not significantly affected in PS2Tg2576 mice at any age examined (Figures 1(d)–1(f)). Thus, PS2Tg2576 mice exhibited increased spontaneous physical activity with regard to some spontaneous behaviors in an age-dependent manner, beginning at 6 months of age. To further confirm the motor performance, we tested rotarod test in PS2Tg2576 mice (see Supplementary Figure 1 in Supplementary Materials). These results indicated that motor coordination is intact in PS2Tg2576 mice throughout the age of 12 months.

### 3.2. Early Impairment of Fear Memory in PS2Tg2576 Mice

**Figure 2:**
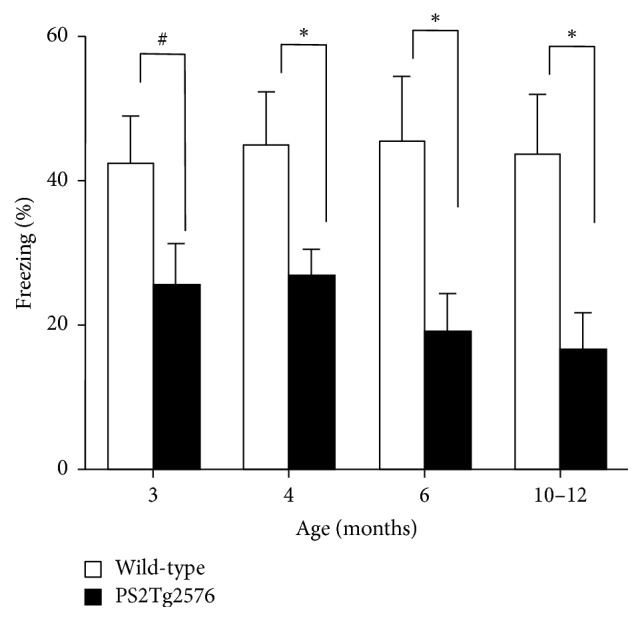
Progressive impairment of contextual fear conditioning in PS2Tg2576 mice with aging. The fear memory 24 hr after footshock was evaluated in PS2Tg2576 mice (closed column, *n* = 10) and their littermate control mice (open column, *n* = 10). PS2Tg2576 mice display impaired memory for context after fear conditioning beginning as early as 4 months of age, with a trend for an impairment observed at 3 months. Data points represent the mean ± SEM. ^*∗*^*p* < 0.05, ^#^*p* < 0.1 versus the corresponding wild-type group.

We then tested contextual fear memory in the control and PS2Tg2576 mice at 4 different stages of aging (Figure 2). In this task, mice learned to associate the environmental context (fear conditioning chamber) with an aversive stimulus (mild foot shock; US), enabling testing for hippocampal-dependent contextual learning ability. In the training trial, there was no significant difference in the distance traveled between the PS2Tg2576 and wild-type mice at any age examined (Supplementary Figure 2 in Supplementary Materials). Test trials were performed 24 h after the training trials. Two-way ANOVA revealed a significant main effect of genotype (*F*_(1,72)_ = 22.446; *p* < 0.0001); however, the effects of age (*F*_(3,79)_ = 0.267; *p* > 0.05) and the interaction effect were not significant (*F*_(3,79)_ = 0.323; *p* > 0.05). PS2Tg2576 mice tended to perform worse than controls beginning at 3 months of age (*p* = 0.071), with the difference becoming significant at 4 months (*p* = 0.041). A significant difference was observed also in 6-month-old mice, with less freezing exhibited by PS2Tg2576 mice than control mice (*p* = 0.021). Contextual memory deficits became more severe in PS2Tg2576Tg mice aged 10–12 months (*p* = 0.012), indicating an age-dependent impairment of contextual fear memory in this mutant line (Figure 2). The time course of the freezing behavior during the test trial also indicated that the significant memory impairments were observed in 4-month old PS2Tg2576 mice compared to wild-type mice (Supplementary Figure 3 in Supplementary Materials).

**Figure 3:**
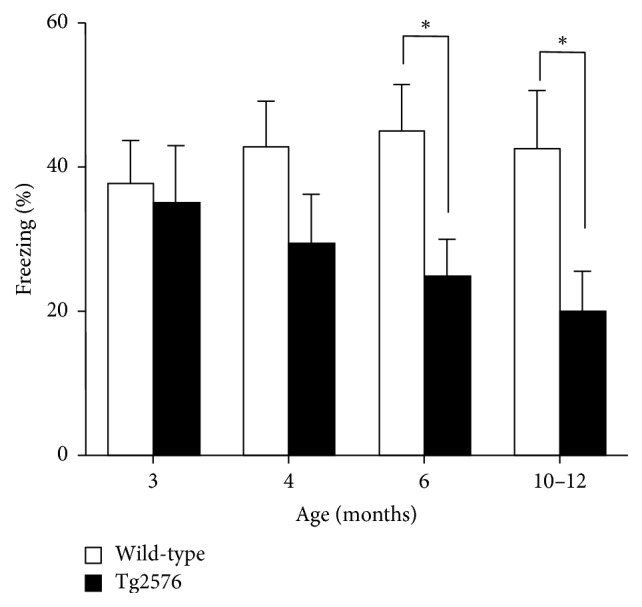
Contextual fear conditioning in the Tg2576 mice. The fear memory 24 hr after footshock was evaluated in Tg2576 mice (closed column, *n* = 10) and their littermate control mice (open column, *n* = 10). Tg2576 mice display intact contextual memory 24 hr later at 3 and 4 months of age. A significant impairment of contextual fear conditioning is observed, with lower levels of freezing to context in 6-month-old Tg2576 mice compared to controls. The impairment is also significant in 10-month-old Tg2576 mice. No differences were observed in freezing to context in control mice across all ages examined. Data points represent the mean ± SEM. ^*∗*^*p* < 0.05 versus the corresponding wild-type group.

Finally, to compare transgenic mice expressing only mutated APP with PS2Tg2576 mice, we investigated when Tg2576 mice exhibited an impairment of contextual fear memory by using the same conditions as those described in the experiment testing PS2Tg2576 mice (Figure 2). We tested contextual fear memory in Tg2576 and their littermate control mice at ages of 3, 4, 6, and 10–12 months (Figure 3). Two-way ANOVA revealed a significant main effect of genotype (*F*_(1,72)_ = 21.29; *p* < 0.001); however, the effects of age (*F*_(3,79)_ = 0.32; *p* > 0.05) and the interaction effect were not significant (*F*_(3,79)_ = 0.441;* p* > 0.05). A significant difference was first observed in 6-month-old mice, with less freezing exhibited by Tg2576 mice than control mice (*p* = 0.025). In contrast, no significant difference in contextual memory was observed in 3- and 4-month-old Tg2576 mice (*p* > 0.05) (Figure 3). In the training trial, distance traveled was not significantly different between the PS2Tg2576 and wild-type mice at any age examined (Supplementary Figure 2 in Supplementary Materials).

## 4. Discussions

The present study demonstrates early-onset cognitive deficits of contextual fear conditioning in an AD mouse model expressing the human mutant* APP* and* PS2* transgenes (PS2Tg2576 mice). PS2Tg2576 mice exhibited significant impairment of fear memory as early as 4 months of age, with a trend towards impairment observed at 3 months (Figure 2). However, spontaneous behaviors and motor coordination were not significantly altered in PS2Tg2576 mice before the age of 4 months, even though some activities, such as walking, of PS2Tg2576 mice tend to be augmented at 4 months of age (Figure 1).

In contrast, Tg2576 mice, which express only human mutant* APP*, did not show cognitive impairments in fear conditioning until the age of 6 months (Figure 3). This result is consistent with that of several previous reports [15, 17, 32], but one previous report has indicated that contextual fear conditioning was impaired in Tg2576 mice as early as 4 months of age [16]. Taken together, the present results suggest that* PS2* mutation accelerates the onset of cognitive impairment of contextual fear conditioning.

Another major finding in the present study was that PS2Tg2576 mice exhibited increased spontaneous physical activities from the age of 6 months (Figure 1). The increase in spontaneous activity in these AD model mice was reported by several previous studies [28, 33, 34]. The phenomenon might be associated with wandering, which is often a common issue in patients with AD [34]. In the case of Tg2576 mice, increased spontaneous activities were observed from the age of 10 months in the home-cage environment [28]. Thus, the current study indicates that* PS2* mutation also accelerates the onset of the alteration in spontaneous physical activities in an AD mouse model. The present finding also supports the hypothesis that decreased freezing behaviors observed in PS2Tg2576 mice at the age of 4 months (Figure 2) are attributable to learning deficits, and not to abnormalities in motor performance.

Identification of the genetic risk factors for AD (missense mutations in* APP*,* PS1*,* PS2*, and* APOE*) has enabled the production of a transgenic mouse model of AD [12, 35, 36]. Among these genes, the presenilin genes harbor approximately 90% of the mutations linked to early-onset FAD, and* PS2* mutation is responsible for approximately 5% of FAD [36, 37]. A substantial number of studies have been devoted to the evaluation of cognitive or behavioral abilities in AD model mice expressing human mutant* PS1* and* APP* [17, 18, 33, 38]. Furthermore, conditional inactivation of* PS1* prevents amyloid accumulation and rescues contextual fear memory impairments in APP transgenic mice [39]. Thus, accumulating evidence indicates that* PS1* mutation accelerates the onset timing of cognitive deficits. In contrast, only few attempts have been made to determine the effect of* PS2* mutation on cognitive performance [21, 22, 39]. A previous study has shown that PS2Tg2576 mice, aged 4-5 months, exhibit cognitive impairments during the probe trial of a MWM task, indicating that* PS2* mutation can accelerate spatial learning impairment [22]. The current result suggesting contextual memory impairment in 4-month-old PS2Tg2576 mice is temporally consistent with the spatial learning impairment (Figure 2).

A substantial number of studies using mouse models of AD have investigated the temporal and causal relationships between A*β* deposition timing and cognitive impairment emergence [40, 41]. In PS2Tg2576 mice, the early deposition of A*β* was observed as early as 2-3 months, but significant accumulation was detected from 4 to 5 months, in several brain regions [40, 41]. Thus, the present results indicated that the onset of significant cognitive deficits coincides with the emergence of significant A*β* accumulation in PS2Tg26576 mice. Compared with many other mouse models of AD, PS2Tg2576 mice manifest progressive AD-like pathology and cognitive dysfunction quite rapidly. For example, in Tg2576 mice or PS1Tg2576 mice, A*β*-containing neuritic plaques appear approximately between 6 and 8 months of age [11, 41–43]. The present study indicates that cognitive impairment in PS2Tg2576 mice could be detectable as early as 4 months of age. Contextual fear conditioning, using PS2Tg2576 mice, could be useful for studying the molecular basis of cognitive dysfunction in AD and for screening new drug/therapy candidates for AD.

## 5. Conclusion

The results of the contextual fear conditioning test presented in the current study demonstrate an age-dependent cognitive impairment in a double-transgenic APP/PS2 mouse model of AD. Compared with the behavioral data on Tg2576 APPswe mice, the present data provides evidence of an accelerator role of PS2 on cognitive impairments in APP transgenic mice. The present study also showed that PS2Tg2576 mice exhibited increased spontaneous physical activities from the age of 6 months. These results could help understand the functional role of PS2 and the relationship between neuropathological events and cognitive/behavioral decline in AD.

## Supplementary Materials

Supplementary Materials Supplemental Figure  1. Rotarod test in the PS2Tg2576 mice. Supplemental Figure  2. The distances traveled by the PS2Tg2576 (upper panel) and Tg2576 (lower panel) mice during the training trial (3 min before shock) of contextual fear conditioning. Supplemental Figure  3. The time course of the freezing behavior during the test trial of contextual fear conditioning. 
