# Supplementary material for: Early Contextual Fear Memory Deficits in a Double-Transgenic Amyloid-β Precursor Protein/Presenilin 2 Mouse Model of Alzheimer's Disease
Source: Int J Alzheimers Dis. 2017 Nov 27;2017:8584205. doi: 10.1155/2017/8584205 (PMC5733185; doi:10.1155/2017/8584205)
Supplement: Supplementary Materials — Supplemental Figure 1. Rotarod test in the PS2Tg2576 mice. Supplemental Figure 2. The distances traveled by the PS2Tg2576 (upper panel) and Tg2576 (lower panel) mice during the training trial (3 min before shock) of contextual fear conditioning. Supplemental Figure 3. The time course of the freezing behavior during the test trial of contextual fear conditioning. [file 8584205.f1.docx]

**Optional Supplementary Materials**

**Methods**

**The rota-rod test**

The rota-rod (Ugo Basile, Comerio, Italy) consisted of a gritted metal roller (3 cm in diameter). Each mouse was placed on a roller rotating at 20 rpm, and the time for which it remained on the rotating roller was measured for a maximum of 120 s. The mice received 5 daily sessions (2 trials per session).

**The distance traveled during contextual fear conditioning**

The movie data of the mouse behavior during the training trial of contextual fear conditioning were analyzed with the HomeCageScan system (CleverSys, Inc., Reston, VA, USA). The distance traveled for 3 min was evaluated.

**Supplemental Figures**

**
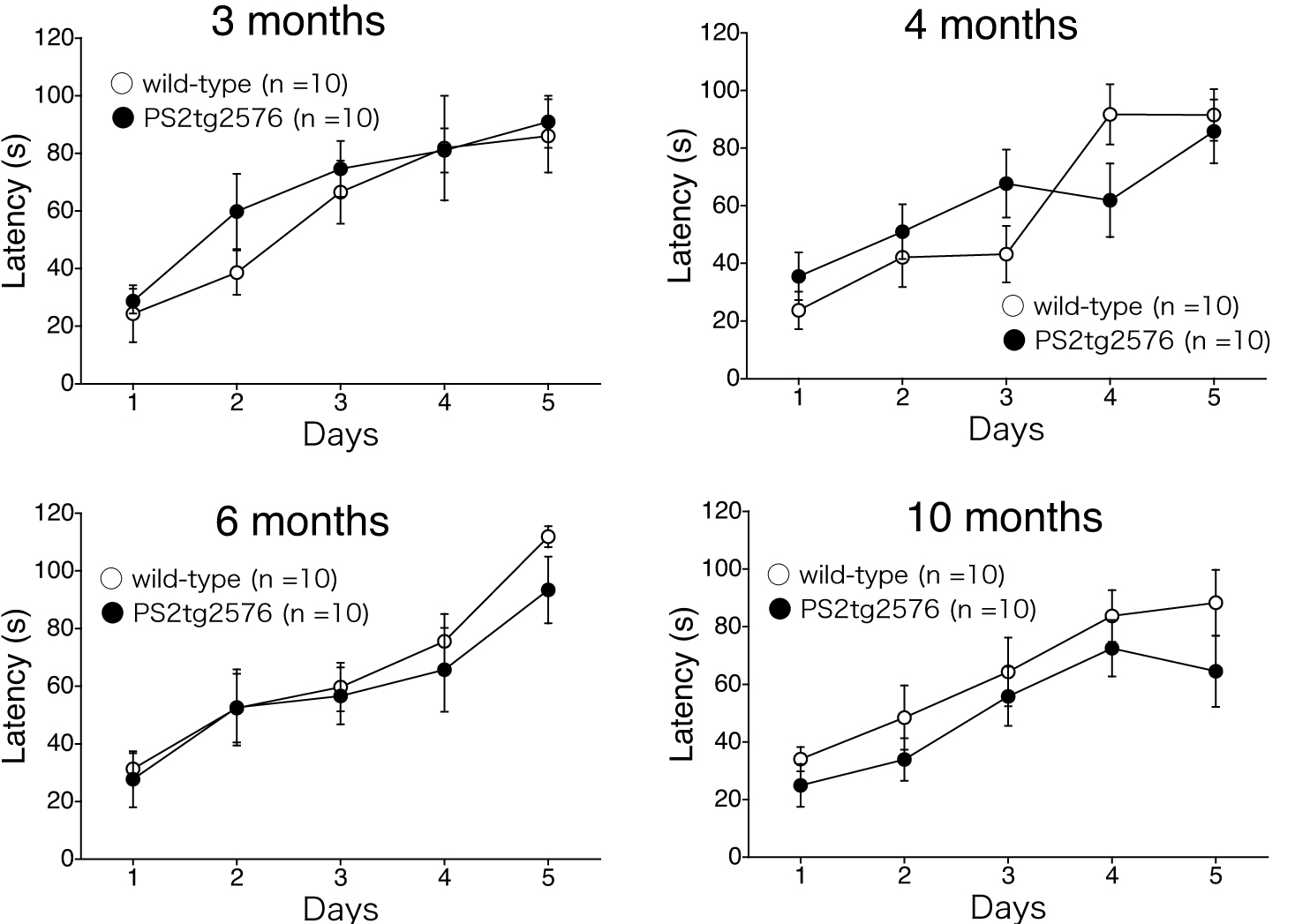
**

**Supplemental Figure Legend 1:** **Rota-rod test in the PS2Tg2576 mice.**

The rota-rod test in the PS2Tg2576 mice (filled circle) and control wild-type mice (empty circle) at ages 3, 4, 6, and 10 months. There was no significant difference in the performance between wild-type and the PS2Tg2576 mice at any age. Data points represent the mean ± SEM.

**
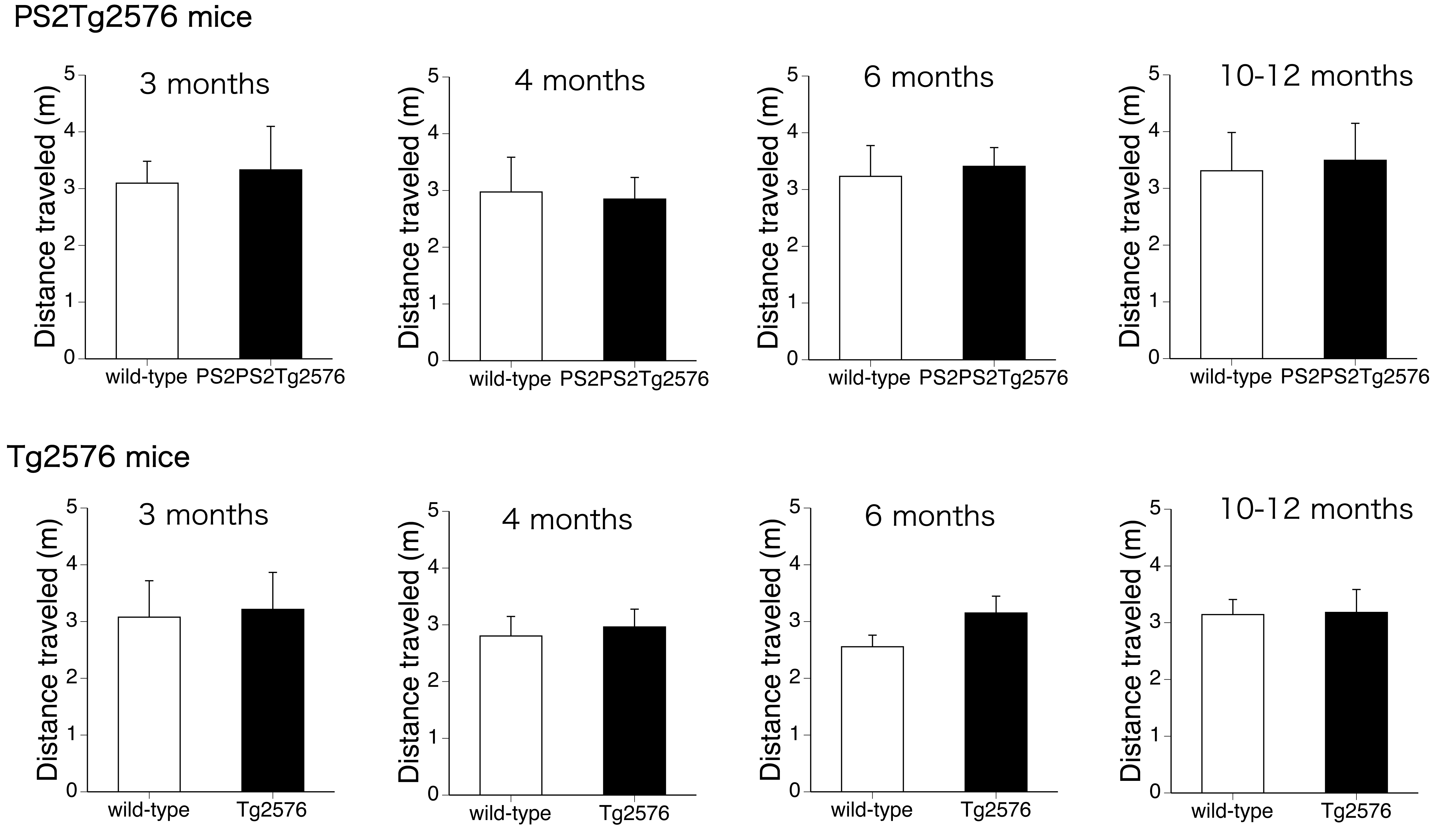
**

**Supplemental Figure Legend 2:** **The distances travelled by the PS2Tg2576 (upper panel) and Tg2576 (lower panel) mice during the training trial (3 min before shock) of contextual fear conditioning.**

There were no significant differences in the distance traveled between wild-type and the Tg2576/PS2Tg2576 mutant mice at any age. The data points represent the mean ± SEM.

**
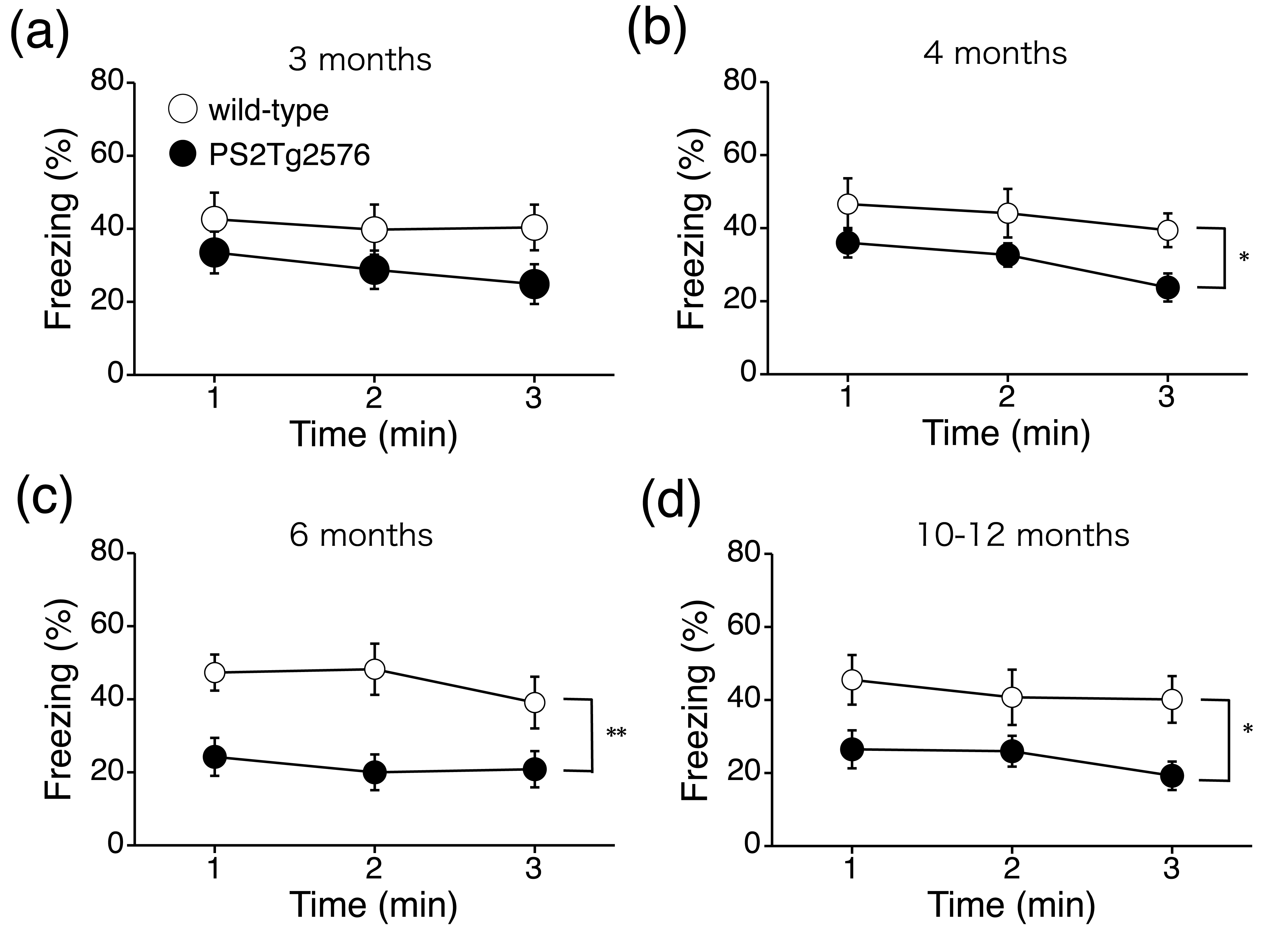
**

**Supplemental Figure Legend 3: The** **time course of the freezing behavior during the test trial of contextual fear conditioning.**

The freezing behavior of the PS2Tg2576 mice was evaluated by each 1-min block during the test trial (3 min). (a) Freezing behavior in wild-type (n = 10) and the PS2Tg2576 (n = 10) mice at the age of 3 months. (b) Freezing behavior in wild-type (n = 10) and the PS2Tg2576 (n = 10) mice at the age of 4 months. (c) Freezing behavior in wild-type (n = 10) and the PS2Tg2576 (n = 10) mice at the age of 6 months. (d) Freezing behavior in wild-type (n = 10) and the PS2Tg2576 (n = 10) mice at the age of 10–12 months. ANOVA revealed a significant difference in the behavior between wild-type and PS2Tg2576 mice at ages 4, 6, and 10–12 months. The data points represent the mean ± SEM. **p* < 0.05, ***p* < 0.01 versus the corresponding wild-type group.
